# Effects of plantar-sensory treatments on postural control in chronic ankle instability: A systematic review and meta-analysis

**DOI:** 10.1371/journal.pone.0287689

**Published:** 2023-06-27

**Authors:** Xiaomei Hu, Jingjing Liao, Xiaoyue Hu, Ziwei Zeng, Lin Wang

**Affiliations:** Key Laboratory of Exercise and Health Sciences, Ministry of Education, Shanghai University of Sport, Shanghai, China; Università Telematica degli Studi IUL, ITALY

## Abstract

**Objective:**

This study aimed to examine the effects of plantar-sensory treatments on postural control in individuals with chronic ankle instability (CAI).

**Methods:**

This study was registered in PROSPERO (registration number CRD42022329985) on May 14, 2022. An extensive search was performed in Pubmed, Embase, Cochrane, Web of Science, and Scopus to identify the potential studies on plantar-sensory treatments affecting postural control before May 2022. The methodological quality of involved studies was assessed using the scale of Physiotherapy Evidence Database (PEDro). The Cochrane Tool and the Risk of Bias in Non-randomized Studies of Interventions assessment tool were used to evaluate the risk of bias in randomised controlled trials (RCTs) and non-RCTs respectively. RevMan 5.4 was utilised to calculate the standardised mean difference (SMD), with 95% confidence interval (CI).

**Results:**

Eight RCTs with a mean PEDro rating of 6 and four non-RCTs with a mean PEDro rating of 4.75 were included in the quantitative analysis. The types of plantar-sensory treatments included plantar massage, whole-body vibration and textured surface-stimulation treatment. A significant effect of static balance with eyes open (SMD = −0.54; 95% CI: −0.81 to −0.27; *p* < 0.001) was found and subgroup analysis showed that plantar massage (SMD = −0.49; 95% CI: −0.84 to −0.14; *p* = 0.006) and whole-body vibration (SMD = −0.66; 95% CI: −1.12 to −0.19; *p* = 0.005) had positive effects. In the subgroup analysis of anterior dynamic balance, whole-body vibration revealed a significant increase (SMD = 0.60; 95% CI: 0.06−1.14; *p* = 0.03). The pooled results or subgroup analysis including eyes-closed static balance and other directions of dynamic balance indicated no significant difference (*p* > 0.05).

**Conclusions:**

This meta-analysis indicated that plantar-sensory treatments could improve postural control in CAI, especially the treatments of plantar massage and long-term whole-body vibration.

## 1 Introduction

Lateral ankle sprain is one of the most common musculoskeletal injuries sustained during physical activity [1, 2]. Up to 40% of those who have experienced lateral ankle sprain may develop chronic ankle instability (CAI), represented by long-term pain, swelling, ankle joint instability, the sensation of ‘giving way’, and functional reduction [3, 4]. CAI can be categorised as mechanical ankle instability (MAI), functional ankle instability (FAI) or recurrent ankle sprain on the basis of pathogenesis and symptoms [5, 6]. Symptoms of MAI are pathological laxity, joint restrictions and degenerative or synovial changes [7]. However, FAI is often presented as proprioception, neuromuscular control and strength disorders [8]. Additionally, recurrent ankle sprain may coexist with FAI and MAI [5]. These pathological conditions of CAI can damage the quality of life and lead to the risk of post-traumatic osteoarthritis [9].

Many studies have documented sensorimotor deficits in individuals with CAI [6, 10, 11], and these deficits link to postural instability [12, 13]. To date, many interventions aim to improve motor function and proprioception in CAI and they have revealed positive effect on postural instability [9, 14, 15]. However, these enhancements remain insufficient in preventing postural instability in patients with CAI [16–19]. In addition to improving motor function and proprioception in those with CAI, considering further treatments to enhance overall sensorimotor function is critical. The maintenance of postural stability is contingent upon not solely the motor system and proprioception but also other sensory information and central integration [20, 21].

According to research, somatosensory input contributes to 70% of the sensory system in controlling posture [22]. The foot directly interfaces with the ground, and somatosensory information from plantar skin benefits postural control [21]. The plantar skin contains various mechanoreceptors, including Ruffini ending, Pacinian’s corpuscule, Meissner corpuscule and Merkel cells, which can be activated by moderate mechanical stimuli and code the corresponding sensory information [21]. Moreover, plantar-sensory inputs can modify the proprioception of the ankle and enhance the perception of the joint position to make a more rapid and accurate response, which has great importance in preventing sports injuries [21]. However, long-term development from ankle sprain to CAI may induce changes in the spinal cord or cerebral cortex levels, ultimately inhibiting sensory-information transmission from the peripheral mechanical receptors [23]. Consequently, individuals with CAI exhibit diminished plantar sensitivity, whereby the plantar skin fails to accurately relay sensory information, thereby heightening the probability of postural instability [13, 24].

Researchers have attempted to enhance postural control in patients with CAI by stimulating plantar receptors [25, 26]. Initially, LeClaire et al. [25] discovered that a 5-minute plantar massage improved static balance with eyes open in individuals with CAI. Similarly, Wikstrom et al. [26] compared three types of plantar massage, including traditional plantar massage, self-administered massage and sensory brush massage, and reported that all three resulted in comparable improvements in postural control. This finding provides empirical evidence suggesting that the stimulation of plantar cutaneous receptors is responsible for the improvements in postural control [26]. However, MeKeon and Wiksteom [27] suggested that plantar massage had limited ability to enhance static balance with eyes open. Similarly, McKeon et al. [28] indicated that the use of textured shoes to stimulate plantar skin impaired postural control. Therefore, the effect of plantar-sensory treatments, intended to stimulate plantar receptors for improving functional performance in individuals with CAI, on postural stability remains equivocal.

To our knowledge, no study has examined the effect of plantar-sensory treatments on postural control in CAI. Accordingly, this systematic review and meta-analysis aimed to review the current evidence to determine whether plantar-sensory treatments can enhance postural stability in individuals with CAI.

## 2 Methods

### 2.1 Search strategy

This study was enrolled in the International Prospective Register of Systematic Reviews on May 14, 2022 (registration number CRD42022329985) and completed in accordance with the PRISMA checklist. Systematic literature retrieval was conducted by the primary researcher in five electronic databases, namely, Pubmed, Embase, Cochrane, Web of Science and Scopus, to obtain studies that investigated postural-stability alteration in CAI before and after plantar-sensory treatments. An additional search was performed to check the reference list. The search strategy was designed by two authors through discussion and it comprised keywords joined with ‘AND’. The keywords had four parts: (1) ankle-associated words, (2) injury-associated words, (3) posture-associated words, and (4) treatment-associated words [10]. Furthermore, ‘OR’ was applied to link each term in each part. The detailed search approach for PubMed is presented in S1 File.

### 2.2 Eligibility criteria

The inclusion criteria included the following: (1) individuals with CAI identified by questionnaires (e.g. Cumberland Ankle Instability Tool, Foot and Ankle Ability Measure and Foot and Ankle Disability Index) and symptoms, with the exception of those who presented with MAI only. The symptoms should include a history of at least one lateral ankle sprain and at least two episodes of ‘giving way’. (2) The interventions of studies must contain sensory treatments that stimulate plantar receptors by devices, therapeutic techniques or other methods. (3) The control group encompassed control, placebo comparison/sham stimulation or other interventions. (4) The assessments involved static or dynamic balance test. (5) English language, full-text and peer-reviewed human studies. The exclusion criteria were as follows: (1) Physiotherapy Evidence Database (PEDro) scale ratings were lower than 4 points [9], (2) data from the study could not be obtained and (3) study design or results are questionable.

### 2.3 Study selection and data extraction

After the duplicates were removed, the search results were independently assessed by two authors on the basis of titles, abstracts and full-text screening by using the aforementioned eligibility criteria. A third researcher was consulted if any issue could not be resolved through discussion.

A Microsoft Excel (2019) table was used to extract the study design, inclusion/exclusion criteria of literature, sample size, participants’ character, intervention, assessment and results. If the original text did not include the target data or the information was equivocal, an email was sent to contact the corresponding author for help or confirmation.

### 2.4 Quality and risk-of-bias assessment

Two researchers individually utilised the PEDro scale, which was found to be a reliable and valid tool to evaluate the methodologic quality of the included studies [29]. The scale encompasses an 11-item checklist in which each item is marked with ‘yes’ or ‘no’. ‘Yes’ represents 1 point except for the first item, with a total of 10 points [30]. In the PEDro scale, studies scoring 6 or more affirmative answers are considered high-quality evidence, whereas studies scoring 4 or 5 are regarded as ‘fair’ and scoring below 4 is deemed ‘poor’ [29]. The Cochrane Risk of Bias Tool was used to evaluate the bias of randomised controlled trials (RCTs) [31]. Six parts of this tool are available to identify the bias in the individual and the whole. Each part could be noted as low, high or unclear risk. Meanwhile, the bias of non-randomised studies of interventions (NRSI) was detected by the Risk of Bias in Non-randomised Studies of Interventions assessment tool [32]. This tool contains 7 domains and 34 signal questions to comprehensively review the risk of bias [32]. The bias in every domain and overall was marked as low, moderate, serious, critical or no information. Finally, the Grades of Recommendation, Assessment, Development, and Evaluation (GRADE) was used to evaluate the quality of this evidence [33].

### 2.5 Statistical analysis

A meta-analysis of the random-effect model was executed using Review Manager version 5.4 to compute the pooled results and 95% confidence intervals (CIs) to examine the effects of plantar-sensory treatments in CAI. Standardised mean difference (SMD) was used to estimate the effect size (ES), which was determined as small (SMD = 0.2–0.5), moderate (SMD = 0.5–0.8), and large (SMD ≥ 0.8), to reflect the size of the effect of plantar-sensory treatments [34]. Additionally, subgroup analysis was be conducted on the basis of intervention methods. *I*^*2*^ statistic was calculated to determine the heterogeneity. According to the Cochrane Handbook, heterogeneity between 0% and 40% may not be significant, 30%–60% may represent moderate heterogeneity and 50%–90% may indicate significant heterogeneity [35]. The most heterogeneous article was discarded when heterogeneity was substantial (i.e., *I*^*2*^ ≥ 60%) [9]. The risk of publication bias was assessed with a funnel plot. In terms of sensitivity analysis, one-study-removed method was used to test the outcomes’ robustness.

## 3 Results

### 3.1 Study selection and characteristics

**Fig 1:**
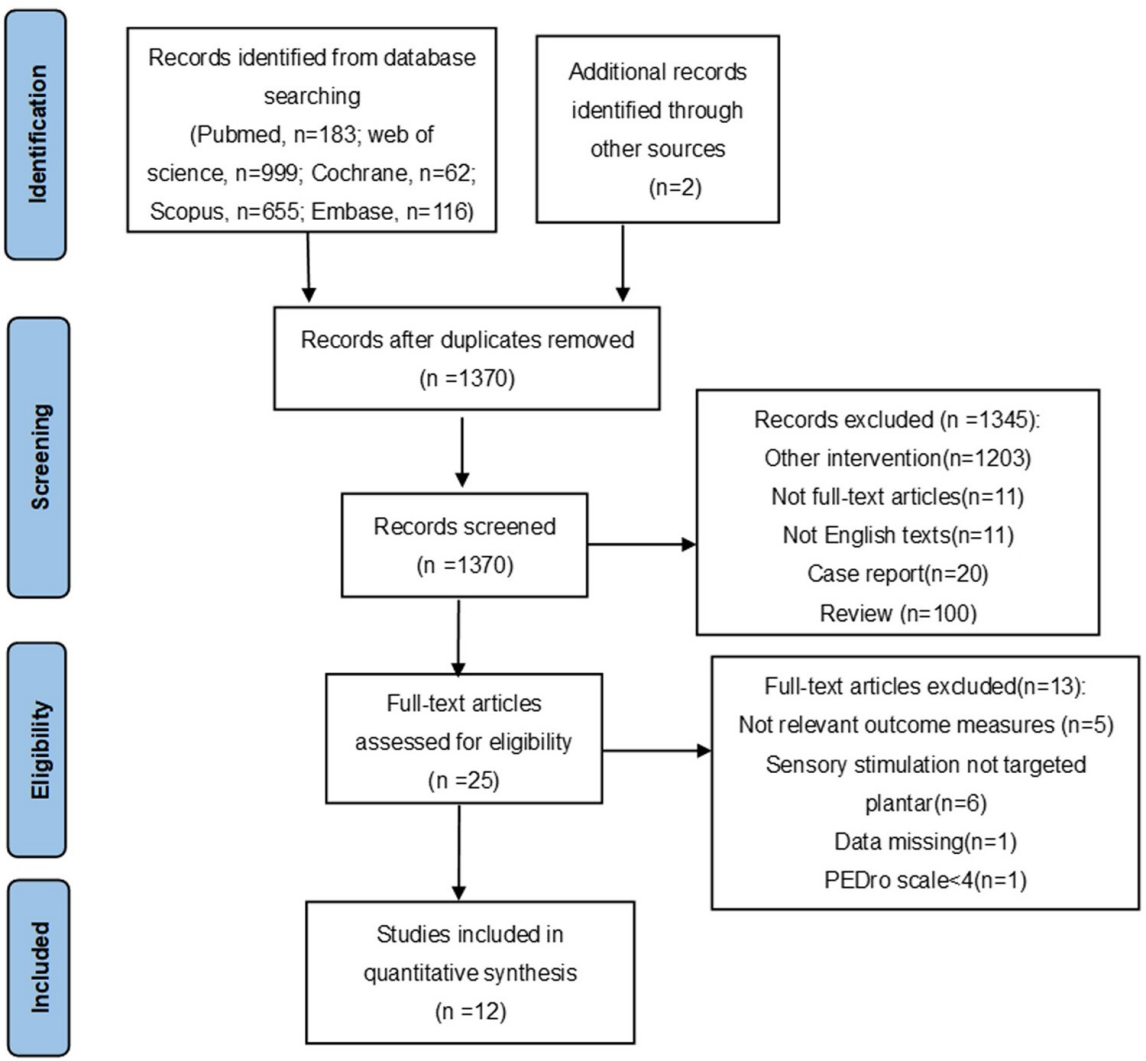
Study selection process.

The flow chart (Fig 1) showed that a total of 2017 potential papers were identified after a systematic search. After 647 duplicates were removed, primary screening was conducted by titles and abstracts and ineligible studies were excluded, 12 studies were included in this analysis.

Amongst these studies, eight were RCTs, three were crossover designs, and one was repeated-measure design. As for the participants, a total of 484 subjects with CAI were included, and 324 individuals were in the quantitative analysis. The participants in the studies by Chang et al. [36] and Cloak et al. [37] included only females. Self-reported questionnaires covering Foot and Ankle Ability Measure, Cumberland Ankle Instability Tool, Ankle Instability Instrument, and Foot and Ankle Disability Index were applied in the included studies to identify the potential individuals with CAI.

The interventions of plantar-sensory treatments included 5-min plantar massage (n = 5), textured surface stimulation (n = 2) and long-term whole-body vibration (n = 5). For the plantar-massage treatment, only two studies by Mckeon and Wikstrom [27, 38] investigated the long-term (i.e. six sessions within 2 weeks) effects on postural control. Shamseddini Sofla et al. [39] investigated the 2-week follow-up effects of whole-body vibration and Sierra-Guzman et al. [40] explored the effect of immediate vibration. The details of the studies included in this meta-analysis are displayed in Table 1.

**Table 1:** Study characteristics.

Author, year study design	Inclusion criteria	Participants and duration	Group and intervention	Assessment
Abbasi, 2019 [41] repeated measure design	**Inclusion**: ankle sprain ≥ 1 (≥ 1 year), ‘giving way’≥2 (≤ 6 months), last ankle sprain ≤ 3 months, CAIT scores < 24. **Exclusion**: history of surgeries or fractures, acute injuries on the lower extremity, anything that influences balance.	30 physical-education students with CAI, both sexes.	WFO: without foot orthosis PFO: prefabricated foot orthosis CFO: custom-molded foot orthosis CTFO: custom-made with textured surface foot orthosis	Immediate **Dynamic balance**: SEBT for three directions (AM, M and PM).
Burcal, 2017 [42] RCT	**Inclusion**: lateral ankle sprain ≥ 2, ‘giving way’ ≥ 1 (≤ 3 months), AII ‘yes’ responses ≥ 4, FAAM ≤ 90%, FAAM-sport ≤ 80%. **Exclusion**: balance or vision problems, acute lower extremities and head injuries (≤ 6 weeks), history of ankle surgeries.	24 patients with CAI, both sexes. 4 weeks, 3 sessions/week.	BT: balance training BTS: combining the same balance training with sensory-targeted ankle rehabilitation strategies.	Immediate, 1-week post-test **Static balance**: SLBT on force plate with eyes open and closed for three 10-second trials, including AP-TTB and ML-TTB. **Dynamic balance**: SEBT for three directions (A, PM and PL).
Chang, 2021 [36] RCT	**Inclusion**: ankle sprain ≥ 1, ‘giving way’ ≥ 1 year, CAIT scores ≤ 24. **Exclusion**: acute ankle sprain, history of surgery and any musculoskeletal diseases of the lower extremities.	63 female athletes with CAI, group A (n = 21) and group B (n = 21). 6 weeks, 3 sessions/week.	Group A: same as group B, but training on a vibration platform, amplitude 5Hz 3mm. Group B: balance training on a balance ball. Group C: no training.	Long-term **Dynamic balance**: SEBT for the eight directions (A, AM, AL, L, M, PL, PM and P).
Cloak, 2010 [37] RCT	**Inclusion**: self-reported unilateral CAI, lateral ankle sprain ≥ 1 (≤ 2 years), the recurrent ‘giving way’, CAIT scores ≤ 23. **Exclusion**: ankle injury (≤ 6 weeks), balance or vestibular disorder, history of lower limb breaks or fractures or surgery, current head injury.	38 female dancer WITH CAI: control group (n = 19), and vibration group (n = 19) in this study. 6 weeks, 2 sessions/week	Control group: daily activities. Vibration group: structured 6-week progressive vibration program training.	long term **Static balance**: SLBT on the pressure mat with eyes open for two 30-second trials, including COP area. **Dynamic balance**: SEBT for 8 directions (A, AM, AL, L, PM, PL, P and M).
Cloak, 2013 [43] RCT	**Inclusion**: self-reported unilateral CAI, lateral ankle sprain ≥ 1 (≤ 2 years), the recurrent “giving way.” CAIT scores ≤ 23. **Exclusion**: ankle injury (≤ 6 weeks), balance or vestibular disorder, history of lower limb breaks or fractures or surgery, current head injury, negative results in the anterior drawer test.	33 male amateur football players with CAI: vibration group (n = 11), and wobble board group (n = 11) in this study. 6 weeks, 2 sessions/week.	Vibration group: same as the wobble board group, but training on a vibration platform and progressive task. Wobble board group: balance training. Control group: no intervention.	long term **Static balance**: SLBT on the pressure mat with eyes open for three 30-second trials, including COM distribution. **Dynamic balance**: SEBT for three directions (A, PM and PL).
LeClaire, 2012 [25] crossover design	**Inclusion**: lateral ankle sprain ≥ 1, ‘giving way’ ≥ 2 (< 6 months), AII ‘yes’ responses ≥ 5. **Exclusion**: acute sprain (≤ 6 weeks), history of lower extremity surgery, lower limb injury (≤ 6 months), neuropathy, and disorders that affect balance.	18 patients with CAI, both sexes. 5 min.	Plantar massage group: massage targeted plantar cutaneous receptors. Calf massage group: massage targeted the entire posterior calf.	Immediate **Static balance**: SLBT on a force plate with eyes open and closed for three 20-second trials, including the COP excursion in anteroposterior, mediolateral and resultant.
McKeon, 2012 [28] crossover design	**Inclusion**: self-reported CAI, ankle sprain ≥ 1, recurrent ‘giving way’, AII ‘yes’ response ≥ 4, FADI < 90%, FADI-sport < 88%. **Exclusion**: acute ankle injury (≤ 3 months), history of lower extremity injury (≤ 12 months) and surgery, peripheral neuropathies, vestibular disorder.	20 patients with CAI, both sexes.	Textured group: wearing textured insole. Sham group: wearing insole with no textured. Control group: no insole.	immediate **Static balance**: SLBT on balance platform for three 10-second trials, including the AP-TTB and ML-TTB directions.
McKeon, 2016 [38] RCT	**Inclusion**: lateral ankle sprain ≥ 1, ‘giving way’ ≥ 2 (≤ 6 months), AII ‘yes’ response ≥ 5, FAAM ≤ 90%, FAAM-sport ≤ 80%. **Exclusion**: acute ankle sprain (≤ 6 weeks), history of lower extremity surgeries, anything that affects sensorimotor function.	80 patients with CAI, both sexes, control group (n = 20) and massage group (n = 20) in this study. 5 min/session, six sessions within 2 weeks.	Control group: no intervention Massage group: 5 min plantar massage. Joint mobilisation group: two 2-min sets of joint mobilisation. Calf Stretching group: two sets of calf Stretching.	Immediate (1 session) and long-term (6 sessions) **Static balance**: SLBT errors that occurred during three 20-second trials on a firm surface with eyes closed.
McKeon, 2019 [27] RCT	**Inclusion**: lateral ankle sprain ≥ 1, ‘giving way’ ≥ 2 (≤ 6 months), AII ‘yes’ response ≥ 5, FAAM < 90%, FAAM-sport < 80%. **Exclusion**: acute ankle sprain (≤ 6 weeks), history of lower extremity surgeries, anything that affects sensorimotor function.	74 patients with CAI, both sexes, control group (n = 18) and massage group (n = 19) in this study. 5 min/session, six sessions within 2 weeks.	Control group: no intervention Massage group: 5-min plantar massage Joint mobilisation group: two 2 min sets of joint mobilisation Calf Stretching group: two sets of calf Stretching	Immediate (1 session) and long-term (6 sessions) **Static balance**: SLBT on a force plate with eyes open and closed for three 10-second trials, including deviation of the COP and velocity in anterior-posterior and medial-lateral directions.
Shamseddini Sofla, 2021 [39] RCT	**Inclusion**: significant ankle sprain ≥ 1, first sprain (≥ 12 months), ‘giving way’ ≥ 2 (≤ 6 months), CAIT scores < 24, FAAM < 90%, FAAM-Sport < 80%. **Exclusion**: history of musculoskeletal surgery and fractures in either or both lower limbs, acute injury limited physical activities (≥ 1 day), potential contraindication of whole-body vibration usage and other diseases that affect balance.	34 patients with CAI, both sexes, control group (n = 10) and WBV group (n = 12) in this study. 4 weeks, 3 sessions/week.	Control group: no intervention WBV group: whole-body vibration, amplitude of 3 mm, frequency increased from 30Hz to 40Hz and duration increased from 35s to 60s. WBV-S group: combine whole body vibration and balance training.	Long-term and 2-week follow-up **Dynamic balance**: modified SEBT for three directions (A, PM and PL).
Sierra-Guzma´n ,2018 [40] RCT	**Inclusion**: ankle sprain ≥ 1, ‘giving way’ ≥ 2 (≤ 6 months), CAIT scores ≤ 24. **Exclusion**: history of lower extremity surgeries or fractures, acute musculoskeletal injury of the lower extremity (≤ 3 months).	50 recreational athletes with CAI, both sexes, no vibration group (n = 16) and vibration group (n = 17) in this study. 6 weeks, 3 sessions/ week.	Control group: no intervention; No vibration group: balance training. Vibration group: combined balance training and whole-body vibration.	long-term **Static balance**: SLBT on Biodex Balance System with eyes open for three 20-second trials, including OSI, APSI and MLSI. **Dynamic balance**: SEBT for five directions (A, AM, PM, PL and M).
Wikstrom, 2017 [26] Crossover design	**Inclusion**: significant ankle sprain ≥ 1, ‘giving way’ (≤ 6 months) and AII ‘yes’ response ≥ 5. **Exclusion**: balance or vision problems, acute lower extremity or head injury (≤ 6 weeks) and history of musculoskeletal surgeries or fractures to either limb.	20 patients with CAI, both sexes. 5 min.	Manual group: a clinician-delivered manual plantar massage Ball group: patient-delivered massage using an off-the-shelf massage ball Brush group: clinician-delivered massage using a sensory brush	Immediate **Static balance**: SLBT on the force plate with eyes open for three 10-second trials, including velocity of COP and 95% area ellipse of COP. **Dynamic balance**: SEBT for three directions (A, PM and PL).

A: anterior, AII: Ankle Instability Instrument, AL: anterolateral, AM: anteromedial, APSI: anterior-posterior stability index, AP-TTB: anterior-posterior time-to-boundary, CAI chronic ankle instability, CAIT: Cumberland Ankle Instability Tool, COM: centre of mass, COP: centre of pressure, FAAM: foot and ankle ability measure, FADI: Foot and Ankle Disability Index, L: lateral, M: medial, MLSI: medial-lateral stability index, ML-TTB: medial-lateral time-to-boundary, OSI: overall stability index, P: posterior, PL: posterolateral, PM: posteromedial, RCT: randomised controlled trial, SEBT: star excursion balance test, SLBT: single-leg balance test.

### 3.2 Outcome measures

Dynamic or static balance assessments were used to reflect the effect on posture control before and after plantar-sensory treatments in the included studies. Single-leg balance test (SLBT) was applied with a force plate (n = 7) and the parameters containing centre of pressure (COP)-related parameters, the centre of mass (COM) distribution and time-to-boundary measures. The Biodex Balance System (n = 1) and single-leg stance error (n = 1) were further applied to measure static postural stability. In terms of dynamic balance testing (n = 7), the star excursion balance test (SEBT) was used. Amongst the eight directions of the test, the anterior, posteromedial, and posterolateral were frequently used.

### 3.3 Quality and risk of bias

The results of methodological quality assessment could be found in Table 2. The included studies’ PEDro scores varied from 4 to 8, with a mean of 5.58. Six studies were fair level, whereas the others were high quality. The risk of bias measured with the Cochrane Risk of Bias tool indicated that the overall bias of RCTs may be considered ‘high’ because high biases originating from blinding methods existed. In relation to NRSI, the overall risk of bias could be regarded as ‘moderate’. Amongst these NRSI, only one study had bias owing to missing data [41] Given that the attrition proportion and reason were similar, this domain was considered to have moderate bias. The detailed information on the risk of bias is presented in S1 Fig and S1 Table. GRADE showed that the quality of evidence on single-leg balance with eyes open for single-session plantar massage, long-term whole-body vibration and anterior dynamic balance of vibration were regarded as very low, moderate and low, respectively. Details are presented in S2 Fig.

**Table 2:** PEDro ratings for included studies.

Author, year	0	1	2	3	4	5	6	7	8	9	10	Total score
Abbasi, 2019 [41]	Yes	No	No	Yes	No	No	No	Yes	No	Yes	Yes	4
Burcal, 2017 [42]	Yes	Yes	Yes	Yes	No	No	No	No	No	Yes	Yes	5
Chang, 2021 [36]	Yes	Yes	Yes	Yes	No	No	No	Yes	No	Yes	Yes	6
Cloak, 2010 [37]	Yes	Yes	No	Yes	No	No	No	No	Yes	Yes	Yes	5
Cloak, 2013 [43]	Yes	Yes	Yes	Yes	No	No	No	No	No	Yes	No	4
LeClaire, 2012 [25]	Yes	No	No	Yes	No	No	No	Yes	Yes	Yes	Yes	5
McKeon, 2012 [28]	Yes	No	No	Yes	No	No	No	Yes	No	Yes	Yes	4
McKeon,2016 [38]	Yes	Yes	Yes	Yes	No	No	No	Yes	No	Yes	Yes	6
McKeon,2019 [27]	Yes	Yes	Yes	Yes	No	No	No	Yes	No	Yes	Yes	6
Shamseddini Sofla, 2021 [39]	Yes	Yes	Yes	Yes	Yes	Yes	Yes	No	Yes	Yes	Yes	8
Sierra-Guzma´n,2018 [40]	Yes	Yes	Yes	Yes	No	Yes	Yes	Yes	No	Yes	Yes	8
Wikstrom, 2017 [26]	Yes	Yes	No	Yes	No	No	No	Yes	Yes	Yes	Yes	6

0: eligibility criteria; 1: random allocation; 2: concealed allocation; 3: baseline comparability; 4: participants blinding; 5: therapists blinding; 6: assessors blinding; 7: follow-up > 85%; 8: intended treat; 9: between-group comparisons; 10: point measures and variability.

### 3.4 Data analysis

#### 3.4.1 Static balance

**Fig 2:**
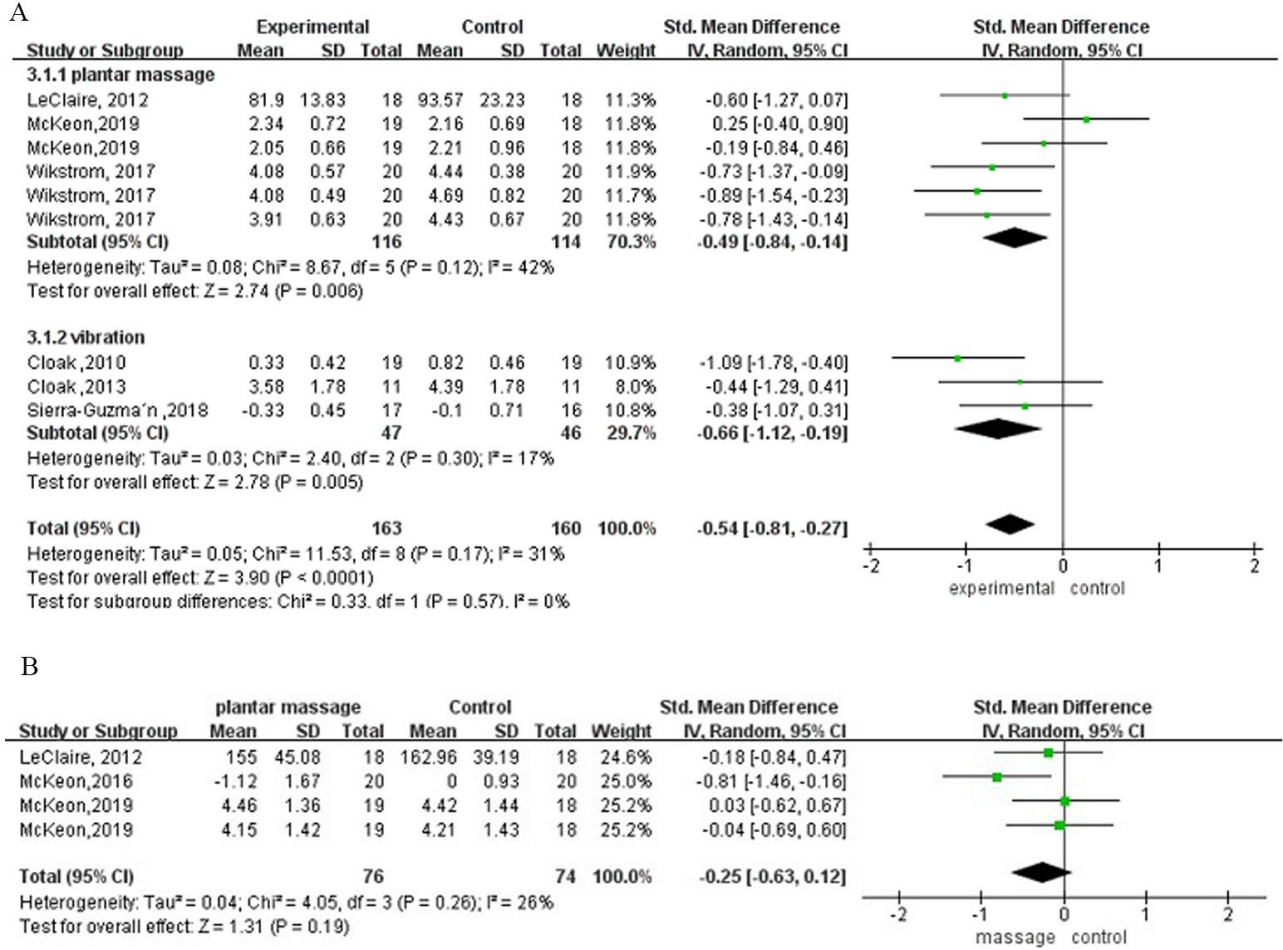
A—, Plantar-sensory treatments versus control group on static balance with eyes open. B—, Plantar-sensory treatments versus control group on static balance with eyes closed.

The random-effects model meta-analysis of static balance with eyes open is illustrated Fig 2A. Five studies estimated the overall balance performance with eyes open, showing a significant effect (*p* < 0.001, ES = 0.54). The subgroup analysis was consistent with the total pooled outcomes. The SMD was −0.49 (95% CI: −0.84 to −0.14) for single-session plantar massage, −0.66 (95% CI: −1.12 to −0.19) for long-term whole-body vibration and −0.54 (95% CI: −0.81 to −0.27) for overall. No significant heterogeneity was found in the subgroup of vibration (*I*^2^ = 42%, *p* = 0.12), whereas moderate heterogeneity was observed in the subgroup of plantar massage (*I*^2^ = 17%, P = 0.30). The funnel plot suggested no publication bias amongst these studies (S3 Fig). The sensitivity analysis demonstrated that the result was reliable. Static balance with eyes closed (Fig 2B) indicated no significant difference for plantar massage (*p* = 0.19).

#### 3.4.2 Dynamic balance

**Fig 3:**
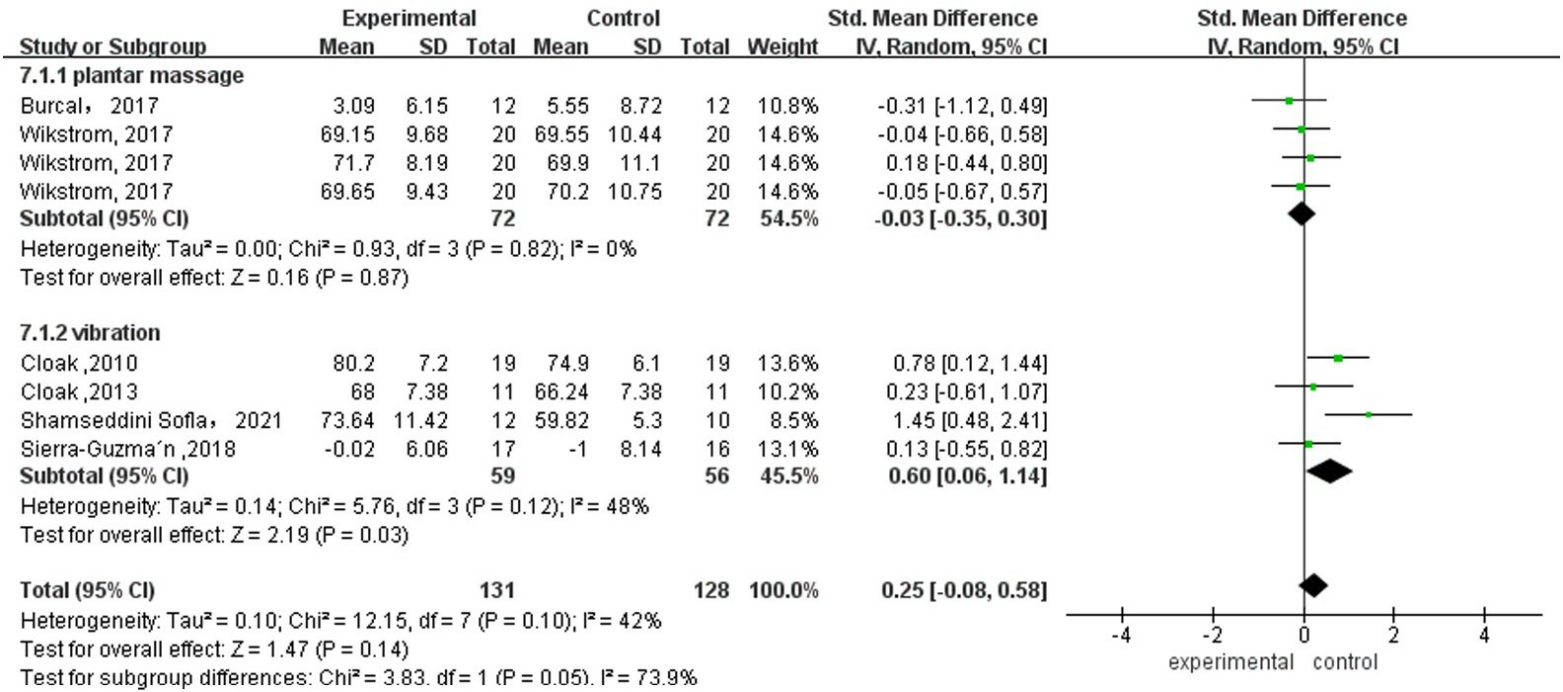
Plantar-sensory treatments versus control group in the anterior direction of dynamic balance.

Seven studies investigated anterior, posterolateral and posteromedial dynamic balances, with three in the medial and anteromedial directions and two in the rest of the directions. All directions presented no significant difference (*p* > 0.05) but the subgroup analysis in the anterior direction showed that long-term vibration had a significant effect (*p* = 0.03, ES = 0.60, Fig 3). The funnel plot showed bias caused by the study of Shamseddini Sofla et al. [39] (S3 Fig).

## 4 Discussion

This systematic review and meta-analysis pooled data from individuals with CAI to examine the effects on postural control before and after plantar-sensory treatments. To some extent, the quantitative results indicated that plantar-sensory treatments could slightly enhance postural stability in CAI. A single bout of 5-min plantar massage improved static balance with eyes open. Long-term whole-body vibration contributed to dynamic balance in the anterior direction and static balance with eyes open. Considering the insufficient studies, the effects of textured stimulation, immediate vibration and long-term plantar massage remained unclear.

The pooled outcomes showed that single-session plantar massage (ES = 0.49) could enhance static balance with eyes open. This finding agreed with previous reviews on CAI [25, 26]. In a narrative review, Helly et al. [44] indicated that plantar massage is beneficial for static balance in CAI. In our study, quantitative evidence was synthesised to make the finding more reliable. Perry et al. [45] suggested that cutaneous sensation on the plantar surface of the foot plays an important role in walking. Besides, Wikstrom et al. [26] reported that plantar massage could stimulate plantar cutaneous receptors rather than underlying musculotendinous receptors. These findings implied that plantar massage may activate plantar cutaneous receptors in individuals with CAI. Activation of plantar cutaneous receptors through plantar massage generates more sensory input, facilitating the central nervous system’s (CNS) detection of changes in movement and production of an effective sensorimotor response to perturbations [46]. However, research investigating the potential of plantar massage to activate plantar receptors and enhance plantar-sensory sensitivity in individuals with CAI is insufficient. Therefore, further empirical research is required to substantiate these claims.

However, plantar massage may not be beneficial to static balance with eyes closed and dynamic stability in individuals with CAI, possibly because the eyes-closed and dynamic tasks were more challenging than the eyes-open one. Song et al. [47] revealed the presence of visual reliance in individuals with CAI, referred to as reweighting of sensory information [22]. The CNS adapts to the diminished proprioception and plantar sensation in patients with CAI by dynamically increasing the weighting of visual input to maintain postural stability [48]. Song et al. [49] reported that reduced plantar cutaneous sensation induced by an ice submersion procedure caused eyes-closed postural control impairments in those with CAI, indicating the ability to dynamically reweight amongst sensory inputs to maintain postural stability appears to be diminished in CAI. The visual reliance and limited capacity to reweight sensory input in individuals with CAI may hinder the effectiveness of plantar massage in improving posture, especially when it is outweighed by the postural instability caused by occluded vision [44]. Similarly, plantar massage was found ineffective in enhancing dynamic balance. As postural control necessitates coordination amongst multiple systems, particularly during dynamic tasks, it presents great challenges. Therefore, a single session of plantar massage is insufficient to make an improvement. Additional treatments (e.g. balance training or strength training) may be necessary to achieve better outcomes in performing more complex motor tasks, such as SEBT, by surpassing mere preparation of sensory system.

With regard to whole-body vibration, the results revealed that vibration had a moderate advantage for static balance with eyes open (ES = 0.66) and dynamic balance in the anterior of SEBT (ES = 0.60). Tan et al. [33] partially supported these findings. They reported that combining whole-body vibration and balance training could enhance postural control abilities. In the present study, the effects of vibration on postural control were explored and further investigation on static and dynamic balance was conducted, indicating that whole-body vibration benefited postural stability in patients with CAI. Vibration is considered to be a common method of activating low-threshold cutaneous mechanoreceptors [50, 51]. It could activate sensory receptors, such as plantar cutaneous mechanoreceptors and musculocutaneous receptors, which cause activation of the ɑ motor neuron pathway, resulting in the contraction of active muscles and the relaxation of antagonist muscles, with simultaneous increase in motor units [52]. However, these changes may be a cumulative effect, as Otzel et al. [53] reported that immediate vibration did not cause changes in motoneuron function. Additionally, whole-body vibration stimulates not only plantar cutaneous receptors but also mechanoreceptors in other joints. The long-term treatment and wider range of vibratory stimulation make it more effective than plantar massage. The funnel plot revealed biases in the study by Shamseddini Sofla et al. [39] possibly due to participants with severe impairment (S2 Table) who showed larger postural improvements before and after treatment.

For long-term plantar massage, only two studies examined the effect of 2-week (i.e. six sessions) plantar massage on static balance. Owing to the limited number of studies and these two studies using different measures, the outcomes could not be synthesised [38]. MeKeon and Wikstrom [27] found that single-session and long-term plantar massage could improve static balance in individuals with CAI but after 72 h, the effectiveness vanished, indicating that the effects of plantar massage may be transient. This finding provides a direction for future research. Besides, only two studies investigated the effect of textured stimulation on postural control, yielding inconsistent conclusions. Abbasi et al. [41] suggested that custom-moulded, textured foot orthosis could contribute to dynamic balance compared with no textured orthosis. Conversely, McKeon et al. [28] implied that textured shoes had a negative effect on mediolateral static balance. Thus, this domain requires further research.

To our knowledge, this meta-analysis was the first to research the effect of plantar-sensory treatments on postural control in individuals with CAI. However, this work has several limitations. Firstly, only a small number of studies were involved, which may potentially affect the results. Secondly, the methods of intervention differed, particularly for vibration, and dissimilar protocols may cause potential bias and restrict the clinical usage. Thirdly, several potential studies were excluded owing to lack of data or low qualities and the included studies were restrained to English language articles, which may lead to selection and publication bias. Finally, the methodological quality assessment showed limited blinding methods that may produce bias. The GRADE recommendations implied only moderate evidence of static-balance enhancement after vibration treatment. Plantar massage could improve postural stability in patients with CAI but this finding should be interpreted cautiously.

## 5 Conclusion

Single-session plantar massage and long-term whole-body vibration may enhance static-balance performance with eyes open and vibration improved dynamic postural control in the anterior direction. Further research should determine whether textured plantar stimulation and long-term plantar massage could also enhance postural control ability. More high-quality and large sample-sized RCTs are required to verify the results of plantar massage on postural performance.

## Supporting information

S1 Checklist PRISMA 2020 for abstracts checklist. (DOCX)

S2 Checklist PRISMA 2020 checklist. (DOCX)

S1 File Search strategies. (DOCX)

S1 Fig Risk of bias assessment in RCTs. (DOCX)

S2 Fig Quality of evidence assessed by GRADE. (DOCX)

S3 Fig Funnel plots. (DOCX)

S1 Table Risk of bias assessment for NRSI. (DOCX)

S2 Table CAIT scores among whole body vibration studies in anterior dynamic balance subgroup analysis. (DOCX)
