# Supplementary material for: Effects of plantar-sensory treatments on postural control in chronic ankle instability: A systematic review and meta-analysis
Source: PLoS One. 2023 Jun 27;18(6):e0287689. doi: 10.1371/journal.pone.0287689 (PMC10298754; doi:10.1371/journal.pone.0287689)
Supplement: S1 File — (DOCX) [file pone.0287689.s003.docx]

**S1 Text. Search strategies.**

Databases: Pubmed, Embase, Cochrane, Web of Science, Scopus

Search data: 2 May 2022.

Search strategies for Pubmed:

1. Ankle-associated words: (ankle [MeSH] OR lateral ligament* OR talo* OR tibiofib* OR tibio-fib*) [Titile/Abstract]
2. Injury-associated words: (ankle injuries [MeSH] OR instabilit* OR unstable OR sprain* OR strain* OR tear*) [Titile/Abstract]
3. Posture-associated words: (postural balance [MeSH] OR postural control* OR postural stability OR postural instability OR posture equilibrium* OR postural sway OR single limb stance OR single leg stance OR double limb stance OR double leg stance OR time to boundary OR time-to-boundary OR TTB) [Titile/Abstract]
4. Training-associated words: (vibration [MeSH] OR insole* OR massage OR plantar sensor* OR plantar stimulate* OR tactile OR light touch OR pressure [MeSH] OR physiotherapy OR train* OR exerc*)
5. Search strategy: 1 AND 2 AND 3 AND 4
