# Supplementary figures and images for: Effects of plantar-sensory treatments on postural control in chronic ankle instability: A systematic review and meta-analysis
Source: PLoS One. 2023 Jun 27;18(6):e0287689. doi: 10.1371/journal.pone.0287689 (PMC10298754; doi:10.1371/journal.pone.0287689)

**S 2 Figure. Risk of bias assessment in RCTs.**


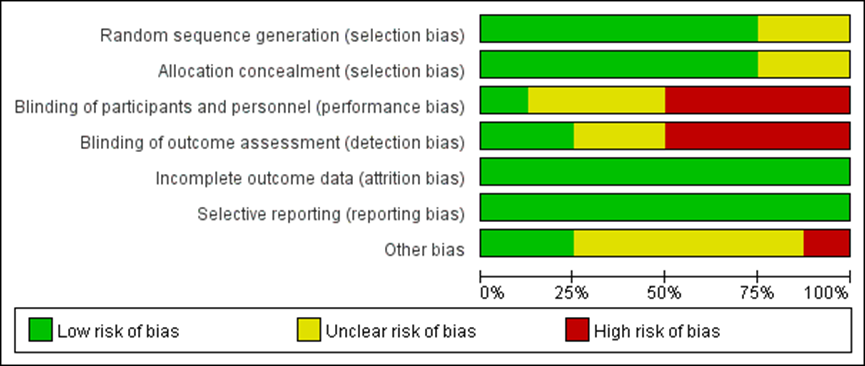


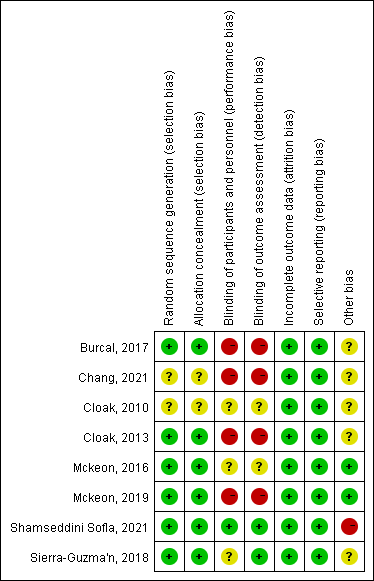

Supplement: S1 Fig — (DOCX) [file pone.0287689.s004.docx]

**S 4 Figure. Quality of evidence assessed by GRADE.**


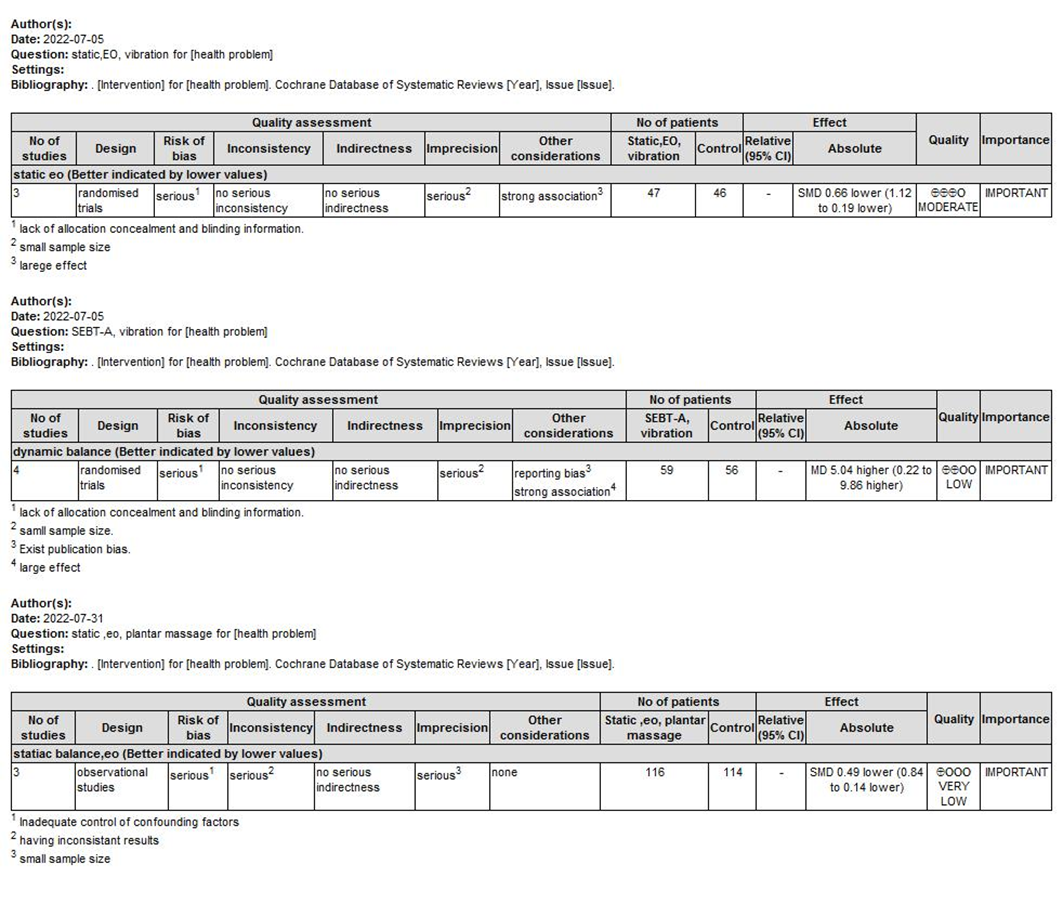

Supplement: S2 Fig — (DOCX) [file pone.0287689.s005.docx]
