# Supplementary material for: Effects of plantar-sensory treatments on postural control in chronic ankle instability: A systematic review and meta-analysis
Source: PLoS One. 2023 Jun 27;18(6):e0287689. doi: 10.1371/journal.pone.0287689 (PMC10298754; doi:10.1371/journal.pone.0287689)
Supplement: S3 Fig — (DOCX) [file pone.0287689.s006.docx]

**S 5. Funnel plots.**


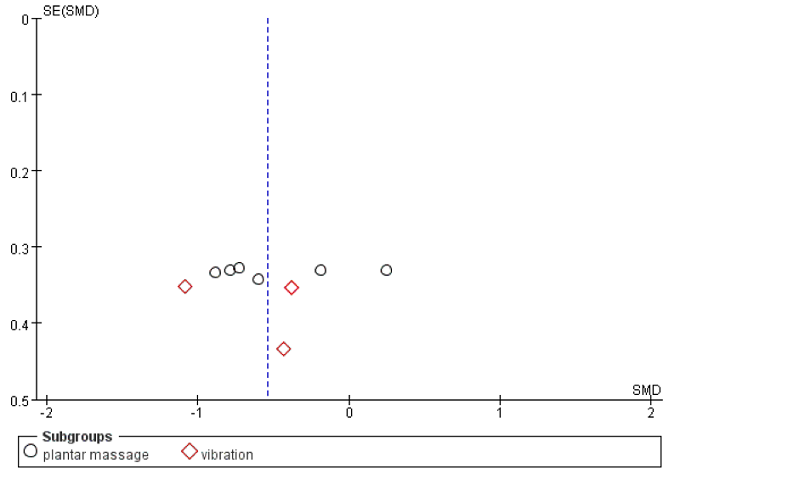


A：Funnel plot showing publication bias amongst studies used to compare plantar-sensory training with the control group in static balance with eyes open.


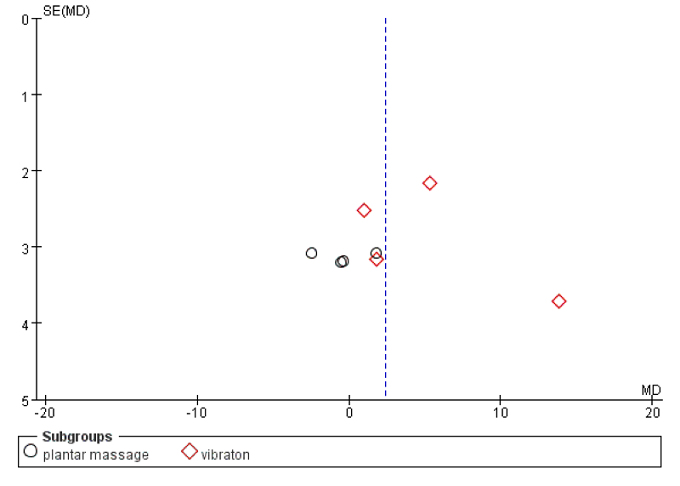


B：Funnel plot showing publication bias amongst studies used to compare plantar-sensory training with the control group in anterior dynamic balance.
