# Supplementary material for: Effects of plantar-sensory treatments on postural control in chronic ankle instability: A systematic review and meta-analysis
Source: PLoS One. 2023 Jun 27;18(6):e0287689. doi: 10.1371/journal.pone.0287689 (PMC10298754; doi:10.1371/journal.pone.0287689)
Supplement: S1 Table — (DOCX) [file pone.0287689.s007.docx]

**S 3 Table. Risk of bias assessment for NRSI.**

| Author, year | D1 | D2 | D3 | D4 | D5 | D6 | D7 | Overall |
| --- | --- | --- | --- | --- | --- | --- | --- | --- |
| Abbasi, 2019 | Moderate | Moderate | Moderate | Low | Moderate | Moderate | Low | Moderate |
| LeClaire, 2012 | Moderate | Moderate | Moderate | Low | Low | Moderate | Low | Moderate |
| McKeon, 2012 | Moderate | Moderate | Low | Low | Low | Moderate | Low | Moderate |
| Wikstrom, 2017 | Moderate | Moderate | Low | Low | Low | Moderate | Low | moderate |

D1: Bias due to confounding; D2: Bias due to selection of participants; D3: Bias due to classification of interventions; D4: Bias due to deviations from intended interventions; D5: Bias due to missing data; D6: Bias due to measurement of outcomes; D7: Bias in selection of the reported results.
