# Supplementary material for: Effects of plantar-sensory treatments on postural control in chronic ankle instability: A systematic review and meta-analysis
Source: PLoS One. 2023 Jun 27;18(6):e0287689. doi: 10.1371/journal.pone.0287689 (PMC10298754; doi:10.1371/journal.pone.0287689)
Supplement: S2 Table — (DOCX) [file pone.0287689.s008.docx]

**S 6 Table. CAIT scores among whole body vibration studies in anterior dynamic balance subgroup analysis.**

| Author, year | Group | Scores |
| --- | --- | --- |
| Cloak, 2010 | control | 18±1.5 |
|  | vibration | 18.4±1.3 |
| Cloak,2013 | control | 17.9±1.3 |
|  | Vibration and wobble board | 18.1±0.9 |
|  | Wobble board | 17.4±1.4 |
| Shamseddini Sofla, 2021 | CON | 9.4±4.45 |
|  | WBV | 10.58±3.44 |
|  | WBVS | 10.41±5.45 |
| Sierra-Guzma´n, 2018 | control | 19.8±2.9 |
|  | Vibration | 18.9±3.2 |
|  | Nonvibration | 19.9±4.1 |
